# The Regulation of Integrated Stress Response Signaling Pathway on Viral Infection and Viral Antagonism

**DOI:** 10.3389/fmicb.2021.814635

**Published:** 2022-02-11

**Authors:** Yongshu Wu, Zhidong Zhang, Yanmin Li, Yijing Li

**Affiliations:** ^1^State Key Laboratory of Veterinary Etiological Biology, Lanzhou Veterinary Research Institute, Chinese Academy of Agricultural Sciences, Lanzhou, China; ^2^College of Veterinary Medicine, Northeast Agricultural University, Harbin, China; ^3^College of Animal Husbandry and Veterinary Medicine, Southwest Minzu University, Chengdu, China

**Keywords:** integrated stress response, eIF2α phosphorylation, unfolded protein response, viral replication, host

## Abstract

The integrated stress response (ISR) is an adaptational signaling pathway induced in response to different stimuli, such as accumulation of unfolded and misfolded proteins, hypoxia, amino acid deprivation, viral infection, and ultraviolet light. It has been known that viral infection can activate the ISR, but the role of the ISR during viral infection is still unclear. In some cases, the ISR is a protective mechanism of host cells against viral infection, while viruses may hijack the ISR for facilitating their replication. This review highlighted recent advances on the induction of the ISR upon viral infection and the downstream responses, such as autophagy, apoptosis, formation of stress granules, and innate immunity response. We then discussed the molecular mechanism of the ISR regulating viral replication and how viruses antagonize this cellular stress response resulting from the ISR.

## Introduction

The integrated stress response (ISR) is an intricate signaling pathway in eukaryotic cells that is activated through the phosphorylation of eukaryotic translation initiation factor 2 alpha (eIF2α) in response to different physiological changes and pathological conditions. Activation of the ISR results in the decrease in global protein synthesis and induction of selected genes, such as activating transcriptional factor 4 (ATF4). It is speculated that the ISR’s ultimate destiny is determined by the intensity and duration of stress, the level of eIF2α phosphorylation, and the activation of ATF4 (Pakos-Zebrucka et al., 2016). A pro-survival effect is activated, and short-term stress reconstructs intracellular homeostasis. However, a cellular death program is initiated when cells are exposed to prolonged and severe stress (Harding et al., 2003; Pakos-Zebrucka et al., 2016). It has been well known that viral infection could induce the ISR, but the role of the ISR is still less defined. In some cases, the ISR is a protective mechanism against virus replication, while in other cases, the ISR may be hijacked by the virus to facilitate its replication. In this review, we summarized current knowledge of the molecular mechanism of the ISR with an emphasis on how cells initiate the ISR and the downstream cellular responses, how viral factors modulate the ISR, as well as cell prognosis upon viral infection.

## Overview of the Integrated Stress Response Signaling Pathway

In physiological conditions, eIF2 consisting of eIF2α, eIF2β, and eIF2γ possess phosphorylation and RNA binding sites. eIF2 forms a ternary complex with GTP and Met-tRNAi and then binds the 40S ribosome subunit, resulting in the formation of the 43S pre-initiation complex (PIC) with two small initiation factors (eIF1 and eIF1A) (Aitken and Lorsch, 2012; Lomakin and Steitz, 2013). The PIC is recruited to the 5′methylguanine cap of mRNA through the eIF4F complex, and the latter contains eIF4G and eIF4E. The PIC migrates to the AUG start codon and binds the Met-tRNAi anti-codon, facilitating protein synthesis. AUG recognition causes the arrest of the scanning PIC and triggers the conversion of the eIF2 GDP-bound state *via* gated phosphate (Pi) release and GTPase-activating (GAP) factor eIF5. The eIF2-GDP complex dissociates from the 40S ribosomal complex and transforms to GTP with the help of the eIF2B complex and enters another recycling of initiation of mRNA translation (Jackson et al., 2010; Hinnebusch and Lorsch, 2012). Under stress conditions, phosphorylated eIF2 can fully form an initiation-competent eIF2-TC, phosphorylated eIF2-GDP tightly binds to and sequesters the guanine nucleotide exchange factor eIF2B to abrogate its activity after its release, and most mRNA translation is reduced during eIF2α phosphorylation. However, translation from certain mRNAs with at least two upstream open-reading frames (uORFs) of appropriate type and position can be upregulated, such as ATF4, ATF5, and C/EBP-homologous protein (CHOP). Upregulation of ATF4, ATF5, and CHOP function activates chaperon and protease to promote cellular recovery or activate cellular death pathways under sustained stress.

**FIGURE 1:**
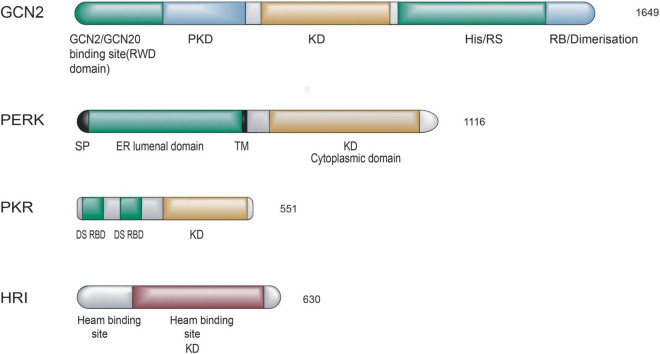
Schematic diagram of the domain organization of the four mammalian eIF2α kinases. Polypeptides are boxes running from N- to C-terminal domains from left to right. Length in amino acids is of proteins. The abbreviations of domains are listed: SP, signal peptide; TM, transmembrane domain; KD, kinase domain; DS RBD, double-stranded RNA binding domain; PKD, pseudokinase domain; His/Rs, histidyl-tRNA synthetase-related domain; RB, ribosome binding. Domains involved in sensing stress signals/activation are in green and black. Kinase domains are yellow and brown, other domains are colored blue, and domains are drawn to scale.

Integrated stress response kinases act as an early responder in mammalian cells to restore cellular homeostasis upon different stimuli. There are four members of the ISR family: general control non-derepressible 2 (GCN2), PKR-like ER kinase (PERK), the heme-regulated inhibitor (HRI), and the interferon (IFN)-induced double-stranded RNA-dependent protein kinase (PKR). Each kinase can sense distinct stresses because each kinase possesses unique regulatory domains, although these kinases share homological catalytic domains (Donnelly et al., 2013; Lavoie et al., 2014). ISR kinases are activated in response to various stress stimuli, GCN2 is sensitive to amino acid starvation, PERK is induced by the accumulation of unfolded or misfolded proteins in the endoplasmic reticulum (ER), HRI is activated in response to heme deficiency, and PKR is activated by double-stranded RNA (dsRNA) (Lussignol et al., 2013). A schematic diagram of the protein structure of mammalian four eIF2α kinases is shown in Figure 1.

Dephosphorylation of eIF2α is a terminal signal of the ISR, and cells return to normal protein translation. The process is mediated by growth arrest and DNA damage-inducible protein (GADD34) that is a constitutive repressor of eIF2α phosphorylation, which interacts with protein phosphatase 1 (PP1) to restore protein synthesis and normal cellular function. GADD34 is a downstream production of eIF2α phosphorylation and ATF4, so GADD34 plays a pivotal role as a negative feedback loop in attenuating ISR signaling (Novoa et al., 2001; Pakos-Zebrucka et al., 2016).

## Virus Modulation of Integrated Stress Response Signaling Pathway

Infection with many viruses can activate the ISR signaling pathway *via* eIF2α kinases. The ISR is generally triggered by virulent or pathogenic viruses instead of inactivated viruses, suggesting that ISR activation is associated with viral replication (Neerukonda et al., 2018). The ISR is mainly induced through PERK/PKR kinases upon single-stranded positive-sense RNA virus infection, and eIF2α phosphorylation inhibits overall protein translation, including viral replication. Furthermore, activation of the ISR initiates downstream signaling such as autophagy, formation of stress granules (SGs), apoptosis, and innate immune response to restore cellular homeostasis. In contrast, viruses can modulate ISR signaling to promote viral replication, activate the ISR signaling pathway, and control viruses upon viral infection, which are summarized in Table 1.

**TABLE 1:** Activation mechanism of ISR signaling pathway upon viral infection.

Type of virus	Virus	Activation of ISR kinases	Activation mechanism	References
A single-stranded positive-sense RNA virus	TGEV	PERK, PKR	TGEV replication is inhibited through activation of NF-κB, which facilitates the production of type I IFN, and the activation of PERK-eIF2α-P inhibits viral replication. Autophagy is activated through PKR upon TGEV infection.	Cruz et al., 2011; Xue et al., 2018
	SARS-CoV	PERK, PKR	Both kinases do not affect viral replication, and the PKR kinase induces apoptosis.	Krahling et al., 2009
	IBV	PERK, PKR	IBV infection causes ER stress and induces PERK and PKR through eIF2α phosphorylation, which activates the expression of ATF4, ATF3, and GADD153. GADD153 exerts its pro-apoptotic activities and promotes viral replication *via* suppressing Bcl2 and antagonizing the survival kinases (ERKs).	Liao et al., 2013
	HCV	PERK	The core protein of HCV induces autophagy, and UPR-induced autophagy promotes viral replication through PERK and ATF6 pathways.	Wang et al., 2014
	EMCV	PERK	2C and 3D protein of EMCV induce autophagy, and UPR-induced autophagy promotes viral replication through PERK and ATF6 pathways.	Hou et al., 2014
	BVDV	PERK	BVDV infection induces the pro-apoptosis process through the PERK-eIF2α pathway, leading to the expression of CHOP, caspase12, and PARP. The influence of viral replication is unknown.	Jordan et al., 2002
	DENV	PERK	PERK is induced at the early stage of DENV2 infection. IRE1a and ATF6 pathways are activated at the late stage, leading to the expression of GADD34 and CHOP, resulting in apoptosis. DENV-induced autophagy promotes viral replication by forming the autophagosome, which provides a dock and energy for viral replication.	Umareddy et al., 2007; Pena and Harris, 2011; Datan et al., 2016; Lee et al., 2018
	WNV	PERK, PKR	The activation of PERK limits WNV replication. PKR is induced at the late WNV infection stage and inhibits viral replication.	Samuel et al., 2006; Medigeshi et al., 2007
	JEV	PKR	JEV infection induces PKR at the late stage. NS2A of JEV promotes viral replication through blocking eIF2α phosphorylation induced by PKR.	Tu et al., 2012
	SINV	PKR, GCN2	SINV infection induces PKR and GCN2 kinases. GCN2 inhibits early viral translation and prevents viral replication through the activation of eIF2α phosphorylation.	Gorchakov et al., 2004; Berlanga et al., 2006; Domingo-Gil et al., 2011
	SFV	GCN2	GCN2 kinase is induced upon SFV infection and inhibits viral replication.	Berlanga et al., 2006
	EV71	PKR	EV71 infection induces typical SGs through the PKR pathway. However, EV71-induced SG-like structures are antiviral RNA granules to suppress viral propagation.	Zhang et al., 2016; Zhu et al., 2016
	PFV	PERK	PFV induces a complete autophagic process through UPRs; increasing activation of autophagy inhibits viral replication.	Yuan et al., 2017
	MNV	PKR	MNV infection induces the PKR pathway through eIF2α phosphorylation. NS3 protein of MNV controls host protein translation. Meanwhile, MNV recruits G3BP1 to promote viral replication and prevent SGs formation.	Fritzlar et al., 2019
Double-stranded DNA virus	HSV	PERK, PKR	PKR is induced firstly, and PERK is activated when viral protein accumulates in the ER. Activation of PERK and PKR phosphorylates eIF2α to block translation of viral protein. However, the γ_1_34.5 protein promotes viral replication by recruiting PP1 to dephosphorylate eIF2α. Us11 and ICP34.5 protein of HSV-1 can block activation of PKR-eIF2α signaling pathway and regulate autophagy by binding directly PKR-binding domain and binding to Beclin1, respectively, to promote viral replication.	Cheng et al., 2005; Lussignol et al., 2013; Zhang et al., 2017
	BTV	PERK	BTV induces ER stress-mediated autophagy *via* the PERK-eIF2α pathway and promotes BTV1 replication.	Lv et al., 2015
A single-stranded circular DNA virus	PCV2	PERK	Cap protein of PCV2 activates UPR-induced apoptosis *via* the PERK-eIF2α-ATF4-CHOP-Bcl-2 axis. Meanwhile, PCV2 can utilize UPR to promote viral replication and expression of Cap protein.	Zhou et al., 2016, 2017
Double-stranded RNA virus	MRV	Unknown	MRV infection induces the formation of SGs at an early phase of infection through eIF2α phosphorylation, and it is speculated that PERK and PKR may play a role in MRV induction of SGs.	Smith et al., 2006; Qin et al., 2009
A single-stranded negative-sense RNA virus	VSV	PKR	VSV infection induces SGs formation through eIF2α phosphorylation; however, TIA1 inhibits viral replication.	Dinh et al., 2013

To date, there is no evidence that the HRI can be activated with virus infection in mammal cells. The HRI of *Epinephelus coioides* (EcHRI), a homolog gene in fish, is changed at the transcription level upon red-spotted grouper nervous necrosis virus (RGNNV) infection and inhibits viral replication through upregulating the expression of IFN-related cytokines, which indicates the potential role of the HRI in antiviral response (Zang et al., 2019).

The study of GCN2 against RNA viruses is not very common. GCN2 was specifically induced through eIF2α phosphorylation at an early stage of sindbis virus (SINV) infection, two non-adjacent regions of SINV genomics bonded to the histidyl-tRNA synthetase-related domain of GCN2 during this process, and GCN2 blocks early viral replication of SINV through eIF2α phosphorylation (Berlanga et al., 2006; Krishnamoorthy et al., 2008).

### Induction of Autophagy Through Integrated Stress Response Signaling Pathway

Autophagy is a conserved cellular lysosomal degradation process and is very important for cell survival and homeostasis. Many studies showed that the activation of unfolded protein response (UPR) regulates autophagy and controls viral replication during viral infection, although the functions between UPR and autophagy are independent. ER transmembrane receptors initiate UPRs: ATF6, inositol-requiring enzyme1 (IRE1), and PERK, a member of ISR kinases (Zhang et al., 2017). PERK-eIF2α-ATF4-ATG12 and IRE1α-JNK-Beclin1 signaling pathways were induced through autophagy to promote viral replication during dengue virus2 (DENV2) infection, IRE1α-JNK released Beclin1 *via* Bcl-2 phosphorylation, which triggered autophagic activity, and the PERK-eIF2α-ATF4-ATG12 signaling pathway partly had an effect on autophagy at the early stage of DENV infection (Lee et al., 2018). Another report showed that PERK participated in DENV-induced autophagy to enhance viral replication by forming autophagosomes in dog madin-darby canine kidney (MDCK) and mouse embryonic fibroblasts (MEFs), which provides a dock and energy for viral replication (Datan et al., 2016). This phenomenon is common in other members of the family *Flaviviruses*, such as hepatitis C virus (HCV), Japanese encephalitis virus (JEV), and West Nile virus (WNV) (Fukasawa, 2010; Syed et al., 2010; Martin-Acebes et al., 2011), which indicates that DENV and other members of the family *Flaviviruses* are ER-tropic viruses that accomplish translation, replication, and package in the ER.

Other viruses also induced autophagy through UPRs to enhance viral replication. Bluetongue virus (BTV) infection induced autophagy through the PERK-eIF2α-ATF4 pathway facilitates viral replication (Lv et al., 2015). Similarly, the duck enteritis virus (DEV) activated autophagy to benefit its replication through the PERK-eIF2α-ATF4 and IRE1-XBP1 signaling pathways (Yin et al., 2017). Autophagy was induced during Newcastle disease virus (NDV) infection promoting viral replication, and P and NP proteins of NDV induced autophagy *via* PERK and ATF6 pathways (Cheng et al., 2016). The core protein of HCV induced complete autophagy, and CHOP played a vital role in UPR-induced autophagy signaling (Ke and Chen, 2011). In addition, UPRs associated autophagy has been found to promote viral replication through PERK-eIF2α-ATF4 and ATF6 signaling pathways, and the activation of ATF4 and CHOP through PERK enhanced the expression of ATG12 and LC3B, which benefits the autophagic process (Tardif et al., 2002; Pavio et al., 2003; Shrivastava et al., 2012). 2C and 3C proteins of EMCV infection induced autophagy through PERK and ATF6 pathways facilitating viral replication (Zhang et al., 2011; Hou et al., 2014). Autophagy was induced *via* ER stress during coxsackievirus (CV) B3 infection, and three branches of UPRs participated in regulating autophagy (Luo et al., 2018). Capsid protein VP2 of the foot-and-mouth disease virus (FMDV) induced autophagy through the eIF2α-ATF4-AKT-MTOR signaling pathway and enhanced FMDV replication by VP2 protein interacting with heat shock protein family B small member1 (HSPB1) in mammalian cells. However, which kinase participates in remains unknown (Sun et al., 2018). These results suggest that viral infection can induce autophagy through UPRs to promote viral replication.

However, prototype foamy virus (PFV) infection induced a complete autophagic process through ER stress containing PERK, IRE1, and ATF6 branches and increased the activation of autophagy to inhibit PFV replication, which implies that PFV-induced autophagy has a novel mechanism and plays an antiviral role in viral replication (Yuan et al., 2017).

Altogether, we concluded that UPRs-induced autophagy facilitates viral replication excepting PFV infection, particularly the PERK-eIF2α pathway. It is speculated that viral proteins may alter ER member morphology and induce the ISR at the early stage of viral infection, phosphorylated eIF2α blocks cellular protein translation, including viral proteins, viral proteins that accumulate in the ER induced prolonged ER stress with a persistent viral infection, and viruses or viral proteins may suppress ER stress-induced cell death *via* modulating UPRs to promote autophagic activity and provide the replication platform and ATP energy for viral synthesis.

**FIGURE 2:**
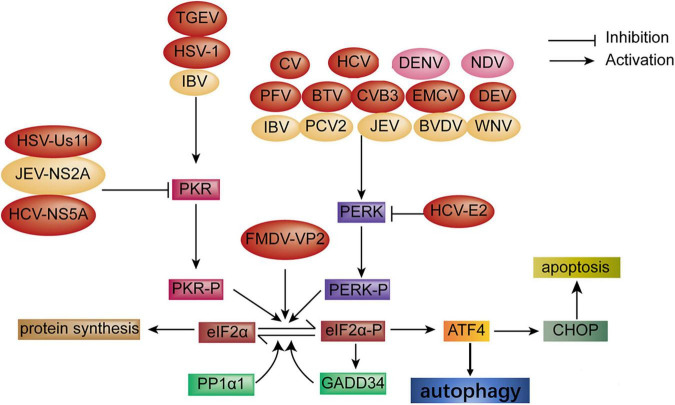
Diagram of the activation of autophagy and apoptosis *via* the ISR signaling pathway during viral infection. Autophagy: Autophagy is activated through the PKR-eIF2α pathway upon infection with transmissible gastroenteritis virus (TGEV) and HSV-1 infection; CV, HCV, PFV, BTV, CVB3, encephalomyocarditis virus (EMCV) and DEV infection, respectively, induces autophagy through the PERK-eIF2α pathway. FMDV-VP2 induces autophagy through interaction with HSPB1 and activation of the eIF2α-ATF4 pathway. In turn, HSV-Us11, HCV-NS5A, and HCV-E2 protein block autophagy (red color). Apoptosis: Apoptosis is induced *via* the PERK and PKR-eIF2α pathway under infection with IBV, PCV2, BVDV, JEV, and WNV, respectively. Oppositely, the JEV-NS2A protein inhibits apoptosis (yellow color). In addition, autophagy and apoptosis simultaneously are induced through the PERK-eIF2α pathway during DENV and NDV infection, respectively (pink color).

PKR was induced by dsRNA through eIF2α phosphorylation and was also required for viral-induced autophagy (Garcia et al., 2007; Lussignol et al., 2013). Autophagy was activated through PKR-eIF2α signaling during herpes simplex virues1 (HSV-1) infection, but the Us11 protein of HSV-1 blocked the activation of PKR-eIF2α signaling by binding directly to the PKR-binding domain (Talloczy et al., 2002; Lussignol et al., 2013), and the ICP34.5 protein of HSV-1 also regulated autophagy through the dephosphorylation of eIF2α and binding to Beclin1 to promote viral replication (Talloczy et al., 2006; Alexander et al., 2007; Zhu and Zheng, 2020). Viral infection activated autophagy through the ISR, and the viral protein hijacking this process is summarized in Figure 2 (red color).

### Activation of Apoptosis Through Integrated Stress Response

Apoptosis, a programming cell death, is a hosting strategy to combat viral infection. The PERK and PKR-eIF2α-ATF4 pathway was activated at the early stage of infectious bronchitis virus (IBV) infection in Vero cells and H1299 cells, which results in the expression of ATF4, ATF3, and growth arrest and DNA damage-inducible153 (GADD153), which is also known as CHOP. Activation of GADD153 induced the ER stress-mediated pro-apoptotic pathway through suppressing Bcl2 and antagonizing the survival kinases (ERKs) that induce tribbles homolog3 (TRIB3) (Liao et al., 2013). However, studies have shown that ER stressor IRE1α was activated in IBV-infected cells and serves as a survival factor during coronavirus infection (Wang et al., 2009; Liao et al., 2013; Fung et al., 2014). HCV triggered apoptosis through the induction of GADD153 and ER calcium depletion (Benali-Furet et al., 2005; Chan and Egan, 2005; Ciccaglione et al., 2005, 2007). JEV infection triggered UPR and apoptosis through GADD153 and p38 kinase expression. However, which branch was induced remains unknown (Su et al., 2002). The Cap protein of porcine circovirus2 (PCV2) induced UPR, resulting in apoptosis through the PERK-eIF2α-ATF4-CHOP-Bcl-2 signaling pathway, which reduces Bcl2 expression and increases caspase3 to enhance viral replication (Zhou et al., 2016, 2017). Some viruses of the family *Flaviviridae* also induced apoptosis *via* induction of a pro-apoptosis response through the PERK-eIF2α pathway, which leads to the expression of CHOP, caspase 12, and poly ADP ribose polymerase (PARP) and the downregulation of Bcl2, such as the NS protein of WNV, bovine viral diarrhea (BVDV), and DENV infection (Jordan et al., 2002; Medigeshi et al., 2007; Umareddy et al., 2007; Pena and Harris, 2011). Three branches of UPRs were involved in NDV-induced apoptosis. Meanwhile, CHOP was initiated by PERK/PKR-eIF2α signaling *via* downregulating BCL-2/MCL-1 to support NDV proliferation (Li et al., 2019). It is speculated that the virus may utilize translational blocking caused by PERK/PKR-eIF2α signaling for the preferential synthesis of viral proteins. Hence, CHOP may serve as a pro-apoptosis or pro-survival function depending on the condition of stress. PERK/PKR signaling pathways were induced in response to severe acute respiratory syndrome-coronavirus (SARS-CoV) infection, leading to sustained eIF2α phosphorylation, which did not inhibit viral replication indicating that SARS-CoV overcame the inhibition of eIF2α phosphorylation through a new mechanism. Furthermore, the activation of PKR induced apoptosis independent of eIF2α phosphorylation (Krahling et al., 2009). UPR-induced apoptosis is summarized in Figure 2 (yellow color).

### Formation of Stress Granules Through Integrated Stress Response

Stress granules are formed by cytoplasmic non-membrane structures of mRNA-binding proteins (mRNPs) and related proteins in response to stress stimuli. It has been proposed that ISR kinases initiate the formation of SGs through eIF2α phosphorylation. However, SGs formation were also independent of eIF2α phosphorylation, such as the disruption of eIF4A helicase by Pateamine A treatment and the eIF4F complex by H_2_O_2_ treatment, which implies that the composition and assembly of SGs differ from that in a stress-dependent manner (Low et al., 2005; Anderson and Kedersha, 2006; Emara et al., 2012). SGs were formed in response to various stresses in mammalian cells, including oxidative stress, energy depletion, UV irradiation, hypoxia, ER stress, and viral infection (Anderson and Kedersha, 2006; Emara et al., 2012; Onomoto et al., 2012).

The virus requires cellular translation machinery to synthesize its proteins in host cells. However, SGs formation results from global translation repression of mRNAs, including the block of viral gene expression during viral infection. Thus, SGs formation may play a role in innate immune response (McCormick and Khaperskyy, 2017). Moreover, the virus also takes measures to confront these adverse conditions and maximizes replication efficiency by inhibiting SGs formation and disrupting processing bodies (PBs) assembly (Zhang et al., 2019). Therefore, the illumination of the relationship between SGs and viruses is very important to understand the interaction of the host and viruses.

The PKR branch of the ISR was activated through eIF2α phosphorylation during murine norovirus (MNV) infection, causing a stoppage of protein translation except the viral replication because MNV can suppress the formation of SGs *via* cytokine translation to promote viral replication, and MNV recruited SGs nucleating protein G3BP1 to enhance viral replication and prevent SGs formation, suggesting that MNV promotes viral replication through the inhibition of SGs formation and evades innate immune response (Fritzlar et al., 2019). Enteroviruses71 (EV71) infection induced the formation of typical SGs (tSGs) *via* the PKR-eIF2α pathway. SGs-like structures were also induced, a different canonical SGs and an antiviral structure to suppress EV71 propagation (Zhu et al., 2016). However, 2A*^pro^* of EV71 blocked tSGs formation and transformed from tSGs to atypical SGs (aSGs) through cleaving eIF4GI. 2A*^pro^* regulated SGs formation, common in picornaviruses (Ye et al., 2018). Several studies demonstrated that the composition of SGs was different from FMDV L*^pro^*, but the exact composition of aSGs remained unclear (White et al., 2007; Zhang et al., 2016; Zhu et al., 2016).

Non-structural protein1-deficient influenza A virus (IAV-NS1^–/–^)-induced cytoplasmic granules are termed antiviral stress granules (avSGs), different from the canonical SGs. IAV-NS1 infection inhibited the formation of avSGs and production of IFN through the PKR-eIF2α signaling pathway (Khaperskyy et al., 2012). SINV, encephalomyocarditis virus (EMCV), Adenovirus, HCV, and NDV also triggered avSGs, implying that avSGs may play an important role in detecting viruses to initiate antiviral signaling. The NS1 protein of IAV blocked the formation of SGs *via* the activation of IFN genes (Onomoto et al., 2012).

Encephalomyocarditis virus was able to transiently induce SGs formation through the PKR signaling at the early stage of infection. However, the 3C protein of EMCV was found to inhibit SGs formation *via* cleaving G3BP1 at the late stage of infection. Similarly, the 3C protein of poliovirus (PV) and the L protein of Theiler’s murine encephalomyelitis virus (TMEV) could inhibit SGs formation (White et al., 2007; Borghese and Michiels, 2011; Ng et al., 2013). These findings indicate that picornaviruses also use the same strategy to evade the immune response by targeting G3BP1, which is essential for the efficient induction of IFN-β.

Hepatitis C virus infection triggered SGs formation *via* the PKR-eIF2α-P signaling pathway (Garaigorta et al., 2012). In addition, SGs formation was induced through the PKR-P-eIF2α-SGs pathway with respiratory syncytial virus (RSV), vaccinia virus (VV), measles virus (MeV) and human immunodeficiency virus (HIV), C protein-deficient Sendai virus (SeV), tick-borne encephalitis virus (TBEV), SINV, EV71, and PV infection (Takeuchi et al., 2008; Heinicke et al., 2009; Venticinque and Meruelo, 2010; Lindquist et al., 2011; Simpson-Holley et al., 2011; Okonski and Samuel, 2013; Yang et al., 2018). It is suggested that the PKR-P-eIF2α-SGs pathway is essential for SGs formation. SGs formation was activated through eIF2α phosphorylation upon reovirus infection. However, which kinase induced this process remains unknown (Smith et al., 2006). Hence, PKR kinase is mainly involved in SGs formation during viral infection, and the formation of SGs plays an important role in antiviral defense and restoring cell homeostasis.

**FIGURE 3:**
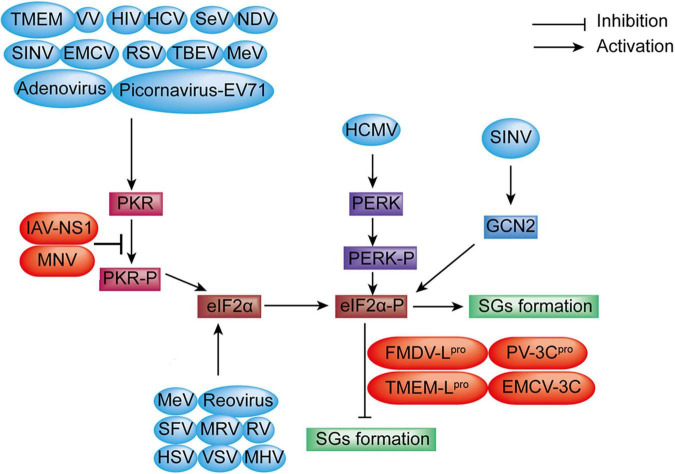
Diagram of SGs formation under viral infection. SGs formation: EV71, VV, HIV, HCV, SeV, EMCV, RSV, TBEV, MeV, TMEV, Adenovirus, NDV, and SINV infection induce SGs formation through the PKR-eIF2α-P signaling pathway. MeV, Reovirus, SFV, rotavirus (RV), HSV, VSV, MHV, and MRV benefit SGs formation *via* direct eIF2α phosphorylation (blue color). However, MNV, nervous necrosis virus (NNV) infection, and the expression of IAV-NS1, FMDV-L*^pro^*, PV-3C*^pro^*, TMEMV-L*^pro^*, and EMCV-3C inhibit SGs formation through blocking eIF2α phosphorylation (red color). SGs formation is increased *via* the PERK-eIF2α-P signaling during human cytomegalovirus (HCMV) infection. SINV infection enhances SGs formation through the GCN2-eIF2α-P signaling pathway.

Recent studies have demonstrated that some viruses induced the formation of SGs at the early stage of viral infection but inhibited the formation of SGs at later stages by blocking the phosphorylation of eIF2α or cleaving SGs scaffold proteins like G3BP1; other viruses inhibited the formation of SGs by altering from SGs proteins to atypical granules to promote viral replication (Raaben et al., 2007; Montero et al., 2008; Qin et al., 2009; Abrahamyan et al., 2010; Rojas et al., 2010; Lindquist et al., 2011; Linero et al., 2011; Ruggieri et al., 2012), such as HCV, RSV, rotavirus, mammalian orthoreovirus (MRV), and mouse hepatitis coronavirus (MHV). Furthermore, SGs formation was induced or inhibited at a different stage of a viral replication cycle or *via* different signaling pathways, such as Semliki Forest virus (SFV), HCV, and RSV (Poblete-Duran et al., 2016), which indicates that it is a game process between SGs formation and antagonism of the virus. The summary of SGs formation through eIF2α phosphorylation during viral infection is shown in Figure 3.

### The Role of Integrated Stress Response in Antiviral Responses

**FIGURE 4:**
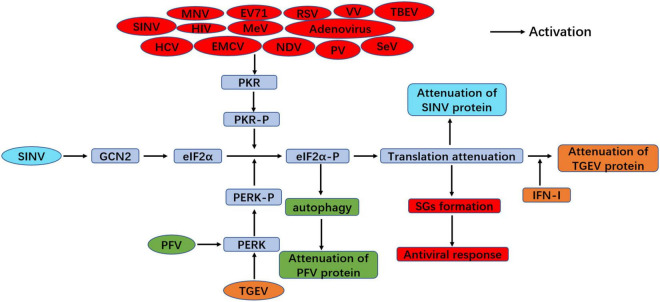
Antiviral response of the ISR during viral infection. PERK-eIF2α signaling suppresses viral replication through inducing IFN-I production and eIF2α phosphorylation-mediated translation attenuation with TGEV infection (orange color). PFV infection inhibits viral replication through PERK-mediated autophagy (green color). In addition, viral infection induces SGs formation through PKR-eIF2α phosphorylation and plays antiviral response, such as MNV, EV71, RSV, VV, TBEV, SINV, HIV, MeV, Adenovirus, HCV, EMCV, NDV, PV and SeV (red color). GCN2-eIF2α signaling inhibit viral replication upon SINV infection (blue color).

Apart from the importance of the ISR on controlling cellular homeostasis, ISR kinases also play a vital role in innate immunity during viral infection, which is thought to function as an antiviral pathway (McCormick and Khaperskyy, 2017; Ma et al., 2018). ISR kinases block overall protein translation through eIF2α phosphorylation, including viral protein. Hence, this process is an antiviral response, and PKR plays an important role in this process because it can directly recruit the formation of SGs. Thus, SGs formation is also an innate immune response to viral infection (McCormick and Khaperskyy, 2017). Transmissible gastroenteritis virus (TGEV) infection was found to activate all three UPRs through the upregulation of GRP78, but the PERK-eIF2α branch mainly suppressed viral replication through inducing IFN-I production and eIF2α phosphorylation-mediated global attenuation of protein translation during TGEV infection *in vitro* and *in vivo* (Xue et al., 2018), which suggests the key role of the PERK-eIF2α-P branch of the ISR in innate immunity. However, another study showed that TGEV infection could only induce the PKR-eIF2α signaling pathway (Cruz et al., 2011). This discrepancy was still unclear, but it might be due to the different TGEV strains used in those two studies. GCN2 also plays a novel role in the antiviral response to certain RNA viruses. GCN2 blocked the translation of viral proteins and further prevented the replication of SINV through eIF2α phosphorylation (Berlanga et al., 2006). The antiviral response of the ISR is summarized in Figure 4.

## Viral Protein Antagonizes Integrated Stress Response Signaling Pathway

The ISR signaling pathway is activated with a different viral infection. Meanwhile, viruses develop different mechanisms to manipulate the ISR signaling pathway to promote viral translation and persistence during viral infection (Ambrose and Mackenzie, 2013b; Green et al., 2014). To facilitate replication, proteins encoded by viruses regulate the ISR pathway selectively and enhance ER protein folding capacity and metabolic regulation of cells. The M protein of vesicular stomatitis virus (VSV) is responsible for counteracting the antiviral response of eIF2α phosphorylation (Connor and Lyles, 2005). The NS5A protein and E2 protein of HCV were found to interfere with PKR and PERK kinase, respectively, which leads to the inhibition of downstream eIF2α phosphorylation and helps viral replication (Pavio et al., 2003; Jheng et al., 2014), whereas the NS2A protein of JEV was found to inhibit PKR-induced eIF2α phosphorylation (Tu et al., 2012; Jheng et al., 2014). IAV-NS1 limited eIF2α phosphorylation through hampering PKR dimerization and autophosphorylation (Lu et al., 1995). Upon DENV infection, PERK-induced eIF2α phosphorylation is suppressed through upregulating the expression of GADD34, which interacts with PP1 to dephosphorylate eIF2α (Pena and Harris, 2011). The protein 7 of TGEV and the M of VSV antagonize eIF2α phosphorylation during viral infection (Connor and Lyles, 2005; Cruz et al., 2011). PERK/PKR was induced through eIF2α phosphorylation at the early stage of HSV infection. However, the γ_1_34.5 protein of HSV inhibited PERK phosphorylation for promoting viral replication. Meanwhile, the expression of GADD34 bonded in an eIF2α-independent mechanism to PP1 to dephosphorylate eIF2α. It is speculated that the γ_1_34.5 protein may recruit PP1 to dephosphorylate eIF-2α and antagonize the activities of both PKR and PERK (Cheng et al., 2005; Zhang et al., 2017). PKR was induced through eIF2α phosphorylation upon MNV infection. At the same time, the expression of IFN-α, IFN-β, and IL-6 was suppressed through eIF2α phosphorylation to promote MNV replication, which is also an immune evasion strategy (Fritzlar et al., 2019).

Generally, viruses hijack cellular protein synthesis mechanisms for the synthesis of viral proteins and disrupt ER homeostasis (Fung et al., 2015). Meanwhile, viruses also develop mechanisms that manipulate the host ISR signaling pathway to promote viral translation and persistence during viral infection (Ambrose and Mackenzie, 2013a; Green et al., 2014).

## Discussion

The ISR is induced through eIF2α phosphorylation by different stresses, leading to the inhibition of overall protein translation and the preferential transcription of targeting genes to restore cellular homeostasis. The ISR was firstly observed in 2002. Recently, the ISR has been concerning because it is a hub for many signaling pathways that converge on eIF2α phosphorylation, which initiates downstream signalings, such as autophagy, apoptosis, SGs formation, cell homeostasis, and innate immunity response.

PERK, an ER kinase, is activated through eIF2α phosphorylation with the accumulation of misfolded and unfolded proteins in the ER. The PERK-eIF2α pathway plays an important role in regulating viral replication *via* UPR-induced autophagy. Other branches of UPRs are also involved, indicating that the synthesis of viral proteins is an ER tropism. We find that UPR-induced autophagy mainly promotes viral replication excepting PFV infection. It is speculated that autophagy has a dual role in regulating viral replication, a survival process is too short to be detected, and a cellular death program is a primary response during viral infection.

PKR plays a vital role in virus-induced SGs formation. It inhibits viral replication through eIF2α phosphorylation and provides a platform to promote the production of the IFN gene (Ruggieri et al., 2012; Albornoz et al., 2014), which means that the formation of SGs is an antiviral response. However, the leader protein of EMCV can inhibit IFN gene activation (Borghese and Michiels, 2011), and some viruses can disturb the formation of SGs (Ng et al., 2013). Altogether, the formation SGs is mainly an antiviral response and provides a platform to inhibit viral replication through the PKR-eIF2α pathway, although the virus confronts this process.

Apoptosis occurs through ISR signaling pathways during viral infection. CHOP-mediated apoptosis is activated through the PERK/PKR-eIF2α signaling pathway for supporting viral replication, which is a complicated process, and other mechanisms are also involved. It is speculated that viruses confront translational shut-off resulting from eIF2α phosphorylation and allow themselves to translate preferentially. However, the role of UPR-mediated apoptosis in viral prognosis needs further elaboration.

Recently, emerging evidence showed that the ISR, autophagy, and apoptosis are induced simultaneously during viral infection (Chiramel et al., 2013; Jheng et al., 2014). It was reported that complete autophagy could be induced during HCV infection. Meanwhile, CHOP played a pivotal role in the ISR-induced apoptosis (Ke and Chen, 2011). The core protein of HCV activated autophagy through PERK-eIF2α-ATF4 and ATF6 pathways to facilitate the expression of ATG12 and LC3 *via* the activation of ATF4 and CHOP (Wang et al., 2014). Hence, the ISR is a complicated and integrated signaling response to different stimuli.

On the contrary, viruses take different strategies for promoting the synthesis of viral proteins. For example, the M protein of VSV can counteract antiviral response. Both Chikungunya virus (CHIKV) and VSV antagonize eIF2α phosphorylation (Connor and Lyles, 2005; Rathore et al., 2013). In addition, some viruses switch translation mode from an eIF2-dependent to an eIF2-independent process to ensure efficient replication, such as PV and enterovirus (EV) (de Breyne et al., 2008; Redondo et al., 2011). It was reported that DENV infection inhibits PERK-mediated eIF2α phosphorylation by elevating the expression of GADD34, which interacts with PP1 to dephosphorylate eIF2α (Pena and Harris, 2011).

As discussed above, the ISR is a complicated, integrated, and adaptational response, and eIF2α phosphorylation is the core of the ISR and blocks overall protein translation to restore cellular homeostasis upon viral infection, suggesting that eIF2α phosphorylation plays an antiviral defense response. However, the decrease in viral proteins resulting from eIF2α phosphorylation is detected at an early stage, and the synthesis of viral proteins is increased at a later stage; it suggests that the ISR is an early sponsor, and the antagonism of virus favoring itself replication is a primary factor at a later stage. A pro-survival response is induced early with short and mild stress to restore cell homeostasis, but cell death signaling is activated at a later stage with prolonged and severe stress during viral infection. However, the shift mechanism between pro-survival and cell death signaling must be further illuminated. It is adverse for viruses with persistent replication, and how virus balances the replication and cell survival for the propagation of progeny.

In conclusion, the role of the ISR is becoming more and more important during viral infection. The ISR is a complicated, integrated, pro-survival cellular response that converges on eIF2α phosphorylation. A PERK-eIF2α signaling pathway is vital in enhancing viral replication *via* UPR-induced autophagy. The PKR-eIF2α signaling pathway involves mainly in the formation of SGs and UPR-induced apoptosis through CHOP expression. Meanwhile, other branches of UPRs are also involved. The virus also modulates ISR signaling pathways to favor its replication, which is vital to illuminating the interaction between the host and viruses and as a therapeutic targeting to enhance host defense against viruses.

## Author Contributions

YmL and YjL conceived the idea and revised and edited the manuscript. YW collected information and drafted the manuscript. YW and ZZ performed the structural analysis. All authors read and made final approval of the manuscript.

## Conflict of Interest

The authors declare that the research was conducted in the absence of any commercial or financial relationships that could be construed as a potential conflict of interest.

## Publisher’s Note

All claims expressed in this article are solely those of the authors and do not necessarily represent those of their affiliated organizations, or those of the publisher, the editors and the reviewers. Any product that may be evaluated in this article, or claim that may be made by its manufacturer, is not guaranteed or endorsed by the publisher.
